# Exclusion of PAX9 and MSX1 mutation in six families affected by tooth agenesis. A genetic study and literature review

**DOI:** 10.4317/medoral.19173

**Published:** 2013-12-07

**Authors:** Victoria Tallón-Walton, Maria C. Manzanares-Céspedes, Patricia Carvalho-Lobato, Ivan Valdivia-Gandur, Sirpa Arte, Pekka Nieminen

**Affiliations:** 1 Human Anatomy and Embryology Unit. Health University of Barcelona campus-Bellvitge, Spain; 2 Biomedical Department and Odontology Department. University of Antofagasta, Chile; 3 Institute of Dentistry. Biomedicum. Universiy of Helsinki. Finland

## Abstract

Objectives: In the present study, it is described the phenotypical analysis and the mutational screening, for genes PAX9 and MSX1, of six families affected by severe forms of tooth agenesis associated with other dental anomalies and systemic entities. Study Design: Six families affected by severe tooth agenesis associated with other dental anomalies and systemic entities were included. Oral exploration, radiological examination, medical antecedents consideration and mutational screening for PAX9 and MSX1 were carried out. Results: No mutations were discovered despite the fact that numerous teeth were missing. An important phenotypical variability was observed within the probands, not being possible to establish a parallelism with the patterns associated to previously described PAX9 and MSX1 mutations. Conclusions: These results bring us to conclude that probably other genes can determine phenotypical patterns of dental agenesis in the families studied, different than the ones described in the mutations of PAX9 and MSX1. Moreover, epigenetic factors can be involved, as those that can reduce gene dosage and other post-transcriptional modulation agents, causing dental agenesis associated or not with systemic anomalies.

** Key words:**Maxillofacial development, tooth agenesis, PAX9 gene, MSX1 gene, gene mutation.

## Introduction

Many terms appear in the literature to describe the congenital absence of a dental organ, being the more accepted classification: hypodontia, defined as the congenital missing of one to five teeth excluding the third molars; oligodontia, as the absence of more than six teeth, excluding the third molars, and anodontia, defined as the complete lack of teeth. These alterations can appear associated with systemic entities and multiorgan syndrome, in isolated, sporadic forms or as an isolated family trait. The congenital absence of one or several dental structures unassociated with complex congenital syndromes is a relatively common dental anomaly. In general, the prevalence of dental agenesis in the European Caucasian populations is situated between 4 and 8% (1,2). In studies carried out in the Spanish population, we find values between 5.6 and 11.4% (3,4). A higher but not significant predominance in females has been reported (3,4). The prevalence of hypodontia of the primary dentition in the European population varies from 0.4 and 0.9%, and a strong correlation between the congenital absence of the primary and permanent dentitions has been reported (5,6).

In most of the studies, excluding the third molars, the lower second premolar is the most frequently missing tooth followed by the upper lateral incisor (7). No clear difference has been found between the maxilla and the mandible, and the left and right sides (7,8).

Several dental anomalies have been reported together with the congenital absence of a dental organ, such as microdontic teeth (9), peg-shaped upper lateral incisors (10), ectopic eruption (10), short roots (11), taurodontism (12) and enamel hypoplasia (10,13) amongst others.

Environmental and genetic factors are related with the failure of odontogenesis (14). Fractures, surgical procedures and other traumas in the dental region have been defined as a cause of the arrest of tooth development (6). Chemotherapy and radiotherapy have been as well related to tooth agenesia (15).

Hundreds of different known genes have been implicated in the regulation of tooth development (16). Numerous different mutations in two transcription factors, MSX1 and PAX9, have been identified in families affected by oligodontia. PAX9 and MSX1 are expressed in the mesenchyme of developing tooth germs, especially at bud and cap stage, as a response to epithelial signals (17). Furthermore, mutations in the B-CATENIN binding protein AXIN2 has been associated with familial oligodontia and a predisposition for colorectal neoplasia (18).

The PAX9, localized in chromosome 14 (14q12-q13), is a member of the PAX family. It has been suggested that this gene establishes the moment and the place of the odontogenesis start (19). Several different mutations in this transcription factor have been identified in families with oligodontia, being affected most of the molars (20-28).

MSX1, a homeobox protein from chromosome 4, is expressed in several embryonic tissues, including the dental mesenchyme. It is related with the regulation of tooth shape and position (19,28). MSX1 gene was associated with congenital missing teeth and different forms of cleft lip/palate or nail dysplasia and complex syndromes (29-31).

Van der Boogaard *et al* (32) have related mutations in the WNT10A with the aetiology of non-syndromic dental agenesis representing a 56% of the cases studied. More recently Al Fawaz *et al*. (33) have found a loss-of function mutation in the SMOC2 gene related to recessive oligodontia in a consanguineous Pakistanese family.

In the present study, we present the analysis of the phenotype and the genotype of six families affected by severe tooth agenesis associated with other dental anomalies and systemic entities, in which mutations of genes PAX9 and MSX1 were not identified.

## Material and Methods

The present study includes subjects from six families affected by severe tooth agenesis associated with other dental anomalies and systemic entities. All of them were patients of the Odontology Service of the Primary Health Centre of Cassà de la Selva (Girona-Spain). According to the regulations of the Ethics Committee of the Institut d´Assistència Sanitària de Girona (Spain), the study was carried out after the collection of the informed consents of the patients or their tutors.

The clinical exploration was carried out by one of the authors of the present study. Panoramic tomograms and photographies were used for the dental analysis of the probands and their relatives. The extraoral pathologies were also taken into consideration. None of the patients and their relatives suffered from a congenital syndromic disease. With this information, we constructed a pedigree of each family.

From gum samples or buccal swabs, the genomic DNA isolation was carried out, for which Qiaamp DNA Mini Kit ® was used according to the manufacturer’s instructions. Samples were taken both from probands and at least three unaffected family members. The primers and sequencing conditions for the genes PAX9 and MSX1 used were as previously described (24). The sequencing results were compared to the sequences previously published, being used the software BLAST2 (http://ncbi.nlm.nih.gov).

## Results

-Pedigree and phenotype analysis.

Five of the probands of the six families under study were females. For these patients, no information about the absence of the primary teeth was obtained.

In the six families under study, third molars were the most common missing teeth, followed by the upper and lower second premolars and the upper lateral incisors. In the proband from Family 2, we observed the absence of the upper and lower lateral incisors and all the third molars. A different pattern was observed in the subject of Family 3 where all upper premolars and both lower second premolar were missing, not being observed absences of incisors or molars. Patients of Families 4 and 5 presented a similar phenotype, being the second premolars and molars the most frequent dental organs affected. In the proband of Family 6, upper and lower second premolars were missing, upper lateral incisors and some molars were also affected. The average number of missing teeth was 8.83, and 6 excluding the third molars ( Table 1).

**Table 1:**
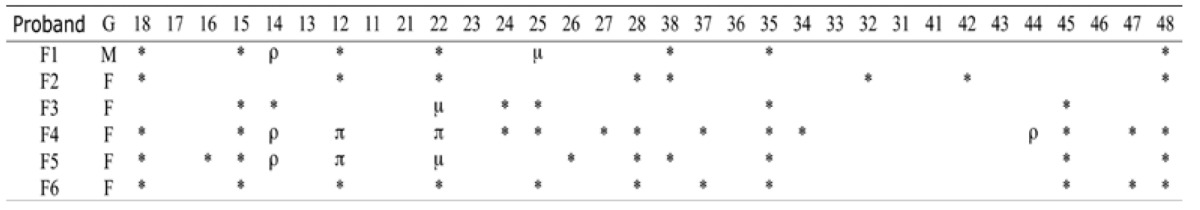
Oligodontia and severe hypodontia phenotypes. G, gender; M, male; F, female; *, tooth missing; μ, microdontia; ρ, rotated teeth; π, peg-shaped teeth.

Some buccal and dental anomalies were observed in these patients. The most common alteration observed were the presence of microdontic teeth, in three of the six subjects under study; being the upper lateral incisors the dental structure more often affected. Rotated teeth were observed in two patients. In addition, two patients presented peg-shaped upper lateral incisors. Pirinen *et al*. (34) described an association between dental agenesia and ectopic maxillary canines; however, this particular dental anomaly that was not observed in any of the probands and their relatives. Mandibular retrognatism was present in two patients, and mandibular prognatism was observed in one of the probands.

If systemic entities were taken into account, allergies and hair anomalies (poor hair) were the most frequent ones. One female patient presented a congenital alteration of a cardiac valve. Another one was affected by Diabetes mellitus. Furthermore, another female patient presented scoliosis.

-Mutation screening of genes PAX9 and MSX1.

The mutation screening for these six patients and their relatives did not reveal the presence of any mutations in the coding regions or in the exon-intron junctions of genes PAX9 and MSX1.

## Discussion

**Figure 1:**
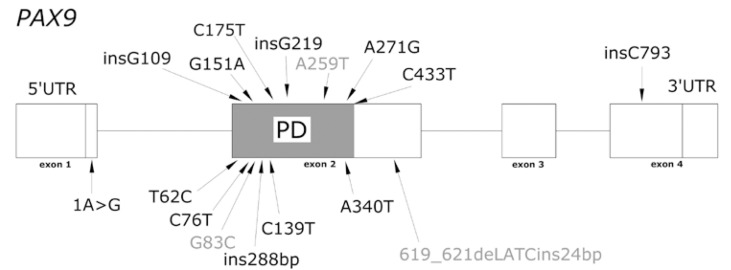
PAX9 MUTATIONS ASSOCIATED WITH DENTAL AGENESIS (PD, paired box): 219insG (20); 340A>T (21); 793insC (45); 271 A>G, 62T>C, ins288pb (23); 83G>C (25); 76C>T (18); 1A>G (46); 109insG, 139C>T (23); 151G>A, 619_621delATCins24bp (26,38); 259A>T (47); 433C>T (48); 175C>T (27).

**Figure 2:**
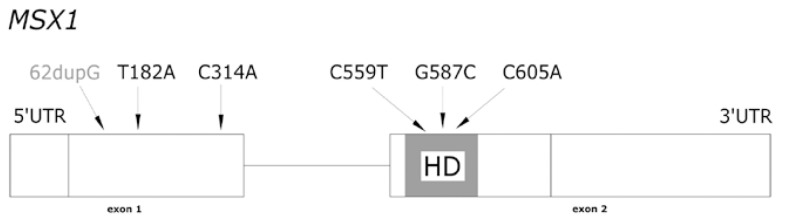
MSX1 MUTATIONS ASSOCIATED WITH DENTAL AGENESIS (HD, homeodomain): 62dupG (44); 182T>A (42); 314C>A (34); 559C>T (43); 587G>C (40); 605C>A, associated with Witkop Syndrom (30).

Up to now numerous mutations and deletions of genes PAX9, MSX1 and AXIN2 had been described associated with tooth agenesis. More specifically fifteen PAX9 mutations had been described associated with severe forms of dental agenesis. Thirteen of them located at the paired box region (exon 2) (Fig. 1). Seven MXS1 mutations have been related with forms of familial tooth agenesis, cleft lip or palate, Witkop and Wolf-Hirschhorn syndromes (Fig. 2). Two mutations in exon 7 of AXIN2 gene have been related with severe forms of tooth agenesis and predisposition to colorectal cancer (18). However, despite the increasing interest in the genetics related to odontogenesis, it is not frequent to find reports of negative results related to the finding of gene mutations in patients with dental agenesis (35-37).

In the six families under study, we did not find any mutations in the coding regions or in the exonintron junctions of genes PAX9 and MSX1, the ones that the literature mentions as related with the aetiology of tooth agenesis (Figs. 1,2). In the same manner, no relation was found between the presence of certain SNPs and these dental anomalies, despite their similar phenotypic characteristics. The phenotypic traits of both the probands and their families were not similar to those described for patients with mutations in the AXIN2 (38).

However, five of the probands selected from the six families under study were females. Many authors indicate a prevalence of congenital missing teeth slightly higher in females (1,39,40).

In family 4, an autosomal dominant form of inheritance seems most probable; in three other families, consanguineous siblings were reported. This inheritance pattern is present in the majority of families affected by non-syndromic oligodontia described in the literature. In the other families, no clear segregation pattern was observed.

Peg-shaped lateral incisors are considered by some authors as a different manifestation of the same genotypes as dental agenesis (4,17). Two female patients presented peg-shaped upper lateral incisors.

**Figure 3:**
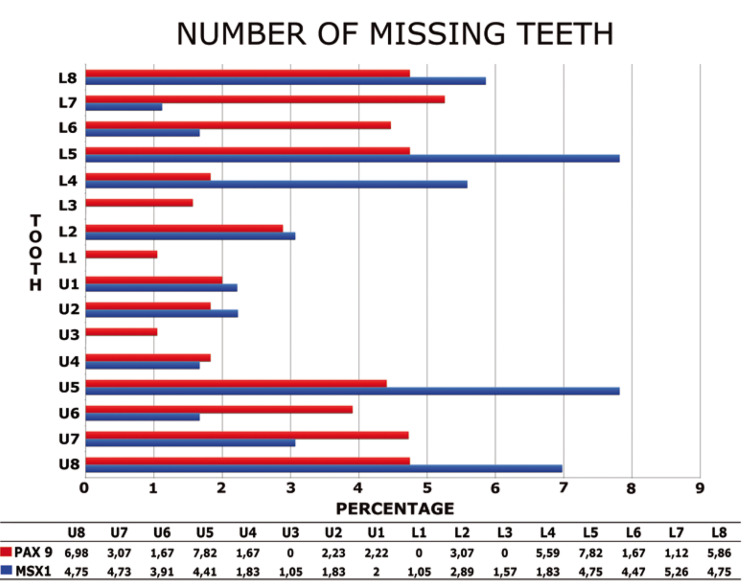
Representation of the missing dental organs in mutations of genes MSX1 and PAX9, cited in chronological order according with its first literature report. Each number represent the average of data about percentage of agenesis obtained for each tooth from different articles. U, upper; L, lower. MSX1 mutations: 41;34;30;42;31;43;44. PAX9 mutations: 20;21;45;22,23;24;26;38;25;46;33;47;48,27.

According to the phenotype of the previously published PAX9 mutations the most often affected teeth were molars and second premolars (Fig. 3). Das *et al*. (22), described the absence of upper lateral incisors and Jumlongras *et al*. of the upper canines and the lower central incisor (25). These characteristics were different from the ones that we could observe in the previously published MSX1 mutations, where the most frequent affected teeth are first and second premolars and lateral incisors (Fig. 3). Consequently, we observed a very different phenotypic pattern between the mutations described in PAX9 and MSX1.

In the six families under study, we could not establish a relation between the phenotypic patterns observed in the mutations of genes PAX9 and MSX1 (Fig. 3), due to the great variability of dental organs missing. These phenotypical differences and the fact that no PAX9 and MSX1 mutations were found in these patients bring us to think that other genes and transcription factors may have an important role in the complex process of odontogenesis and in the aetiology of dental agenesis.

Although we do not find evidence of genetic mutations in the families of this study, alterations associated with epigenetic activity over PAX9 and MSX1 genes can be present. For this analysis, it is necessary to consider that morphological studies of mice modified genetically, indicates that the absence of PAX9 or MSX1 genes in knockout homozygous form can generate various alterations in craniofacial development, including dental agenesis (49,50). Therefore, theoretically the epigenetic regulation should appear at determined times of maxillofacial development in reversible form, causing for example dental agenesis without other associated alteration or as part of syndromic alterations. However, there is little information in the literature about this. Methylation of PAX9 and MSX1 have been associated with cancer development but have not been described the relation between this phenomenon and dental agenesis (51,52). Another study showed evidence that low levels of PAX9 expression, has effects on tooth morphogenesis and generates non-syndromic form of oligodontia in mice (53). This information reveals that epigenetic factors are able to limit the gene expression can generate dental agenesis. Moreover, alterations in the post-transcriptional activity of these genes also may generate dentoalveolar defects. The activity of RNA from MSX1 can be regulated by its own antisense RNA. Therefore, this endogenous molecule would be involved in the regulation of craniofacial development, particularly in the alveolar bone formation (54-56). Another element to consider is the interaction between the PAX9 and MSX1. According to the studies by Ogawa *et al*. (57) and Nakatomi *et al*. (58), a functional relationship between these genes during teeth development has been identified, establishing another potential point of regulation. Thereby, epigenetic intervention directly over DNA or in post-transcriptional activities of one of them, can alter those phases of dental organ development dependents of the mentioned interaction. In addition, these authors suggest that a combined reduction of PAX9 and MSX1 gene dosage in humans may increase the possibility of oligodontia. Moreover, the study of Phillips *et al*. (59) provides valuable information regarding the evolutionary history of PAX9, supporting the hypothesis that post-transcriptional modulation in the expression of this gene could have an effect on the dental formula evolution, suggestion that is supported for the studies realized by Kirst *et al*. (53). Considering our study, it is interesting to note in  Table 1 the high incidence of third molars and second premolars agenesis. This observation is consistent with studies that indicate that progressive reduction in the teeth number was observed in inverse order to how they were formed during development (60,61). More studies are needed to investigate the incidence of epigenetic factors on transcription/translation of MSX1 and PAX9 genes that could trigger non-syndromic dental agenesis in human.

## Conclusions

In the present work, we present the analysis of the phenotype and the genotype of the genes PAX9 and MXS1 of six families affected by severe tooth agenesis associated with other dental anomalies and systemic entities. From the analysis of the phenotypical patterns, a great variability was observed, being not able to establish a parallelism with the ones observed in the previously described PAX9 and MSX1 mutations. The mutation screen of these patients did not reveal the existence of mutations of genes PAX9 and MSX1. These results bring us to conclude that many other genes, such as WNT10A that play an important role during the complex process of odontogenesis are important candidates for the aetiology of tooth agenesis. Moreover, epigenetic factors can be involved, as those that can reduce gene dosage, alter genes interaction and other post-transcriptional modulation agents that also could explain dental agenesis associated or not with systemic anomalies.
